# Dissecting the Human Response to *Staphylococcus aureus* Systemic Infections

**DOI:** 10.3389/fimmu.2021.749432

**Published:** 2021-11-08

**Authors:** Rosanna Leuzzi, Margherita Bodini, Isaac P. Thomsen, Elisabetta Soldaini, Erika Bartolini, Alessandro Muzzi, Bruna Clemente, Bruno Galletti, Andrea Guido Oreste Manetti, Cinzia Giovani, Stefano Censini, Sonia Budroni, Fabiana Spensieri, Erica Borgogni, Silvia Rossi Paccani, Immaculada Margarit, Fabio Bagnoli, Giuseppe Del Giudice, Clarence B. Creech

**Affiliations:** ^1^GSK, Siena, Italy; ^2^Vanderbilt Institute for Infection, Immunology and Inflammation, Vanderbilt University Medical Center, Nashville, IN, United States

**Keywords:** *Staphylococcus aureus*, invasive, antibody, cytokines, isolates

## Abstract

*Staphylococcus aureus* is a common human commensal and the leading cause of diverse infections. To identify distinctive parameters associated with infection and colonization, we compared the immune and inflammatory responses of patients with a diagnosis of invasive *S. aureus* disease to healthy donors. We analyzed the inflammatory responses founding a pattern of distinctive cytokines significantly higher in the patients with invasive disease. The measure of antibody levels revealed a wide antibody responsiveness from all subjects to most of the antigens, with significantly higher response for some antigens in the invasive patients compared to control. Moreover, functional antibodies against toxins distinctively associated with the invasive disease. Finally, we examined the genomic variability of isolates, showing no major differences in genetic distribution compared to a panel of representative strains. Overall, our study shows specific signatures of cytokines and functional antibodies in patients with different primary invasive diseases caused by *S. aureus*. These data provide insight into human responses towards invasive staphylococcal infections and are important for guiding the identification of novel preventive and therapeutic interventions against *S. aureus*.

## Introduction

Staphylococcus aureus is the leading cause of diverse infections that range from uncomplicated skin infections, to more severe diseases, such as pneumonia, sepsis, osteomyelitis, and endocarditis. The spread of methicillin-resistant *S. aureus* (MRSA) strains in both the hospital and community settings has led to greater difficulty in managing staphylococcal disease (1).

*S. aureus* is also a human commensal commonly recovered from the anterior nares, oropharynx, skin and gastrointestinal tract; up to 30% of healthy individuals are persistently colonized with nasal *S. aureus* (2). Several studies demonstrate that disease, as well as colonization, induces both innate and adaptive immune response in the host, though the response to infection is substantially more robust. Upon infection, a proinflammatory response is rapidly elicited with activation of neutrophils, macrophages, and polarized T cell responses, inducing the development of Th1 and Th17 responses. Th1 cells produce IFNγ, a potent innate effector molecule that appears to confer protection against *S. aureus* skin infections and bacteremia (3, 4). Similarly, human Th17 cells and IL-17A/F responses contribute to protective immunity against *S. aureus* infections, particularly against skin, mucosal, and soft tissue infections, promoting neutrophil and monocyte recruitment from the bloodstream to the site of infection (5). To protect against tissue damage, a compensatory, anti-inflammatory response is induced by T regulatory cells that downregulate immune responses by producing anti-inflammatory cytokines such as TGFβ and IL-10. This balance and timing of the pro- and anti-inflammatory responses induced by *S. aureus* bacteremia are predictive of clinical outcomes [reviewed in (6)].

The specific staphylococcal targets recognized by the innate and adaptive human immune response are myriad. Circulating IgG antibodies against several surface antigens and extracellular proteins have been detected in healthy individuals (7), likely as the result of intermittent colonization or very mild staphylococcal infections (e.g., minor skin lesions). Nasal colonization appears to induce specific antibodies and serum antibody levels are higher in persistent carriers than in non-carriers (8, 9). Infection is known to induce a distinctive adaptive immune response, as evidenced by comparisons of antibodies directed to specific *S. aureus* antigens in serum samples of infected patients in comparison to healthy controls (10). Although several studies contributed to our knowledge of bacteremia-specific adaptive immune responses, a major difficulty in defining distinctive antibody pattern in infection and in colonization is the extreme heterogeneity of the individual antibody responses following *S. aureus* infection (11).

In this study we compared the immune and inflammatory responses of 43 patients with a diagnosis of Invasive *S. aureus* disease (12) to 48 healthy donors (HC). The patients had highly variable clinical manifestations including bacteremia, endocarditis, osteomyelitis, and disseminated/multi-focal disease; for each patient the infecting strain was isolated for whole genome analysis.

In an effort to discriminate between antigen-specific antibodies evoked during colonization or invasive disease, we examined antibody responses to a wide antigenic repertoire of 104 antigens. In addition to quantitative assessment of binding antibody, we analyzed the functional antibody response to a number of key virulence factors.

Finally, to characterize the distinctive inflammatory response to invasive disease, we also identified the repertoire of circulating cytokines involved in the acute immune responses.

## Materials and Methods

### Patients and Samples

Study participants were prospectively enrolled at Vanderbilt University Medical Center in Nashville, TN, USA. Serum or plasma samples were obtained from infected patients within 72 hours of culture confirmation of *S. aureus* disease and from healthy, uninfected subjects with no known history of *S. aureus* infection. The VUMC Human Subjects Protection Program (IRB) approved the protocol prior to the initiation of any clinical study procedures. The following subjects were enrolled in the study: 43 patients with invasive *S. aureus* disease (ISA: 18 with bacteremia/sepsis, (3 of which were catheter-associated), 13 with musculoskeletal infection, (3 of which involved prosthetic joints);, 10 with endocarditis, and 2 with pneumonia) and 48 healthy donors (HC) of which 34 were adults and 14 were children. The present study results from the analysis of a subset of the clinical data from patients enrolled at Vanderbilt University Medical Center in Nashville, TN, USA. The samples excluded from the analysis belonged to additional groups that did not reach an adequate number for statistical analyses to extract meaningful biological insight.

### Genomic Analysis

A total of 40 strains from ISA patients were sequenced. Genome sequencing was performed by preparing NGS libraries using the Nextera XT Library Prep kit (Illumina) following manufacturer’s recommendations. TruSeq RAPID, Dual Index, 200bp Paired-End sequencing runs were performed by Illumina HiSeq 2500 NGS sequencer. Isolate assembly was performed through SPADES version 3.9.0. Multilocus sequence typing (MLST) was assessed *via* the BIGSdb platform, which detects and assigns identifiers to specific loci defined on the genomic DNA sequence of each isolate, including identifiers of the 7 MLST genes (as defined on the PubMST database). For whole genome characterization, typing was extended to 2208 core gene loci based on the *S. aureus* PubMLST schema. For the phylogenetic analysis a neighbor net was built with SplitsTree tool version 4.14. A data matrix of MLST+2208 core gene profiles of each isolate was uploaded as input of the analysis. A set of 22 reference *S. aureus* genomes (strains 04_02981, MRSA252, MSSA476, COL, ED133, ED98, JH1, JH9, JKD6008, JKD6159, Mu3, Mu50, MW2, N315, NCTC_8325, Newman, RF122, ST398, TCH60, TW20, USA300_FPR3757 and USA300_TCH1516) representative of the genomic and metabolic diversity of the species were downloaded from GenBank (ftp://ftp.ncbi.nlm.nih.gov/) and introduced into the analysis for comparison.

### Cytokines/Chemokines Assays

Cytokine/chemokine concentrations in samples were measured by V-PLEX Human Cytokine 36-Plex Kit (Meso Scale Discovery) according to manufacturer’s instructions. A total of 81 sera/plasma samples, 39 from ISA and 42 from HC, were tested in two runs. Each sample was tested on four different plates, coated with capture antibodies specific for a different panel of analytes (Chemokine panel: Eotaxin, MIP-1β, Eotaxin-3, TARC, IP-10, MIP-1α, IL-8, MCP-1, MDC; Cytokine panel: GM-CSF, IL-1α, IL-5, IL-7, IL-12/IL-23p40, IL-15, IL-16, IL-17A, TNF-β, VEGF-A; TH17 panel: IL-17A Gen.B, IL-21, IL-22, IL-23, IL-27, IL-31, MIP-3α; and Proinflammatory panel: IFN-γ, IL-1β, IL-2, IL-4, IL-6, IL-8, IL-10, IL-12p70, IL-13, TNF), at 1:2 dilution (Chemokine and Cytokine panels) or 1:4 dilution (TH17 and Proinflammatory panels), according to manufacturer’s instructions. Analytes were analyzed according to pre-set acceptance criteria based on recoveries and CVs of standards and high, medium and low positive controls supplied by Meso Scale Discovery, that were included in each run. Only analytes with valid results in both runs are reported. Concentrations below detection or fit curve range were assigned a value corresponding to ½ of the lower limit of detection (LLOD). The percentage of subjects above and below LLOQ (Lower Limit of Quantitation) and associated two-sided 95% Clopper-Pearson CIs has been summarized by group for each cytokine/chemokine. Difference in the percentage of responders among groups has been evaluated by looking at the p-values of the proportion test in pair-wise comparison between groups with multiple testing correction reported as stars in **Supplementary Table 1**. To compare the level of each analyte among the groups, a nonparametric Kruskal-Wallis (or analysis of variance) with Dunn’s post-test comparison has been performed for each marker. To identify possible separation between ISA and HC and to find out the cytokines/chemokines responsible for any of separation present, principal component analysis (PCA) has been applied. The percentages of subjects showing analyte concentrations above the LLOQ (Lower Limit of Quantitation) in ISA *vs.* HC were compared with the proportion test. Analytes with at least one of the two medians in the quantification range were used in the PCA.

### Protein Chip

A protein array of recombinant *S. aureus* proteins was generated as previously described (13). Briefly, *S. aureus* surface and secreted factors were identified *in silico* using a combined bioinformatics approach and printed on the arrays. The list included 104 antigens, belonging to the *S. aureus* strain NCTC 8325 or Newman, produced in *E. coli* as recombinant proteins and 2 capsular polysaccharides type 5 (CP5) and type 8 (CP8) isolated and purified from *S. aureus* type 5 or type 8 strains; and lipoteichoic acid (LTA, SIGMA). Sera were diluted 1:1000 and analyzed for the detection of total IgG bound to each protein using fluorescently labelled anti-human IgG, measuring the mean fluorescence intensity (MFI) values for each antigen. For each antigen, the IgG titer distribution measured in groups of patients was compared by a nonparametric Kruskal-Wallis test followed by Dunn’s post-test. Euclidean distance has been used, followed by unsupervised hierarchical clustering on antigen reactivities among patients, for descriptive purposes. To identify a set of antigens able to distinguish patients from controls, or subjects from different groups, we have applied a principal component analysis (PCA, data not shown) to select principal components which accounted for the majority of the variation within the dataset. Highly positive sera were selected as the ones with antigen’s concentration above 0.05 ug/ul. Best recognized antigens in tested sera have been detected as those highly positive in at least 10% of the samples in ISA. Test of proportions has been performed on the comparison of the number of highly positive sera for ISA and HC.

### IgG Quantification by Luminex and ELISA

A multiplex bead-based assay (Luminex) was employed to measure total IgG levels against five antigens simultaneously: CP5, CP8, Hla, SpA and ClfA. Luminex microspheres were supplied by Radix BioSolutions. High-Capacity streptavidin magnetic beads 1,25 x 106/ml were conjugated with biotinylated-CPS: CP-5 and CP-8. Hla and ClfA antigens were covalently coupled to MagPlex beads using Luminex standard coupling procedures. Serum samples were serially diluted 1:3 in 96-well microtiter plates in PBS buffer starting from 1:100 to 218,700 (8 dilutions). 50 μL of diluted sera were loaded into a 96-well microtiter plate with 10 μl of antigen coated beads MIX of five antigens (2,000 beads/antigen) and incubated at RT for 90 minutes shaking at 750 rpm. After 2 washes with 100 μL of PBS 1X, 50 μl of an anti-human IgG Phycoerythrin-labeled secondary antibody (Jackson Immuno Research) diluted 1:100 in PBS were added and incubated for 30 min shaking at 750 rpm. The antigen-specific IgG levels were quantified using a Luminex 200 reader.

ELISA was used to measure total IgG levels against LukAB, LukED, and PVL. Purified antigens were diluted in phosphate-buffered saline (PBS) to a concentration of 0.5 µg/mL and bound to 96-well ELISA plates overnight. Wells were then aspirated and blocked at room temperature (RT) for one hour with 5% nonfat dried milk in Tris-buffered saline. Serial 2-fold dilutions of sera were added to the plate and incubated for two hours at RT. After washing, horseradish peroxidase (HRP)-conjugated murine monoclonal antibodies against human total IgG diluted 1:1000 were added, and plates were incubated at RT for 2 hours. Next, substrate solution (3,3’, 5,5’-tetramethylbenzidine) was added and plates were incubated at RT. The reaction was stopped at 30 minutes with 2M sulfuric acid and plates were read spectrophotometrically at 450 nm. Serum depleted of IgG was used as a negative control, and samples were run in duplicate, independently on separate days.

### Analysis of Antibody Functionality

Functional assays for Hla, ClfA, SpA and LukAB were used to measure the neutralizing activity of the sera and plasma samples.

*Hla functional assay*: the ability of antibodies to inhibit Hla-induced hemolysis was evaluated in an *in vitro* rabbit red blood cell (RBC)-based hemolysis neutralization assay, modified from (14). A purified batch of wild-type α-toxin is titrated for its lytic activity by incubation with an equal volume of rabbit RBC dilutions. The RBC dilution giving a hemolysis value ranging between 2.4 and 3.6 at OD405 nm, with a defined concentration of toxin, is determined before each inhibition assay. Serial dilutions of sera or plasma were incubated with 15 nM Hla for 30 minutes at 37°C. Then, RBC derived from defibrinated rabbit blood are added, and incubation is prolonged for a further 30 minutes at 37°C. Each plate included RBC and α-toxin alone as negative and positive control, respectively. Plates were then centrifuged for 5 minutes at 1,000 × g, the supernatant was transferred in a microplate using an automated equipment (Hamilton Nimbus) and then the absorbance was measured at 405 nm by the Bioteck H170 absorbance microplate reader. Each absorbance value was converted in percentage (%) of hemolysis by SoftMaxPro GxP software and a 4-parameter hemolysis dilution curve was obtained. The neutralization titer is defined as the reciprocal serum dilution which neutralizes the toxicity of Hla by 50%.

*ClfA functional assay*: the ability of antibodies to inhibit the binding of ClfA to fibrinogen was measured by an ELISA. Microtiter plates were coated with a purified sub-domain of ClfA wild-type at 10 µg/mL overnight at 4°C. After washes and blocking with 1% BSA -PBS, sera samples were 2-fold diluted and added onto plates followed by incubation for 1 hour at 25°C. Human fibrinogen at 100 μg/mL was added and incubated for 1 hour at 25°C. The plated were then incubated with anti-human fibrinogen/HRP conjugated diluted 1/2,000 for 1 hour at 25°C. After washes, the binding was detected by adding 1-Step TMB ELISA substrate for 15 minutes at 25°C. The reaction was stopped by adding 1M sulphuric acid and the fibrinogen bound to ClfA was evaluated by measuring the absorbance at 450 nm by a PowerWave HT reader (Biotek). Each absorbance value was converted in percentage of binding to fibrinogen by SoftMaxPro GxP software and a 4-parameter binding curve was obtained. ClfA inhibition titer was defined as the serum reciprocal dilution giving 50% binding in reference to a 0% and 100% binding control.

*SpA functional assay:* a Luminex-based assay was designed to measure the ability of specific anti-SpA antibodies to bind to the wild type antigen by their Fab portion, and not *via* the Fc portion. FcγRI (CD64), being expressed by a vast array of human cells, has been chosen for this assay. Luminex beads are coated with the SpA wild-type purified protein and incubated with serially diluted human samples for 30 minutes at room temperature. After washing, biotinylated FcγRI is added for 15 minutes at room temperature. After washing, streptavidin conjugated with Phycoerythrin (SAPE) is added for 15 minutes at room temperature. The mean fluorescence intensity (MFI) measured by a Flexmap3D reader and interpolated in a 5PL logistic calibration curve corresponds to the FcγRI binding to the immuno-complex of SpA protein and anti-Spa antibodies. Results were expressed in relative Luminex Units per mL.

*LukAB functional assays*: Human promyelocytic HL-60 cells (ATCC) were cultured in RPMI 1640 (Cellgro) supplemented with 10% heat inactivated fetal bovine serum (FBS), 100 μg/mL Penicillin and 100 μg/mL Streptomycin (Pen/Strep, Cellgro), and allowed to differentiate to polymorphonuclear-like cells (PMN-HL60) for 3 days with 1.5% DMSO per standard techniques. To measure the toxin neutralization capacity of individual serum samples, serial dilutions of each serum samples were mixed with a fixed amount of purified LukAB (1.25 μg/mL). Samples, in triplicate wells of a 96-well microtiter plate, were incubated for 30 minutes at room temperature. Toxin-only wells served as positive toxicity controls, while media-only wells served as un-intoxicated control. Differentiated HL-60 cells (PMN-HL60) were added (1 x 105 cells/well) to the LukAB and serum wells and incubated at 37°C + 5% CO2 for one hour. The final reaction volume was 100μL. To measure cell viability and metabolism, CellTiter (10 μL/well) (Promega) was added to the wells and incubated for 2 h. Color development was read at 490 nm using a spectrophotometer. The neutralization titer is defined as the reciprocal serum dilution which neutralizes the toxicity of LukAB by 50%.

### Statistical Analysis for IgG Quantification and Functional Assays

For each subject and each antigen tested for IgG quantification through Luminex (ClfA, CP5, CP8, Hla, SpA, LukAB, LukED, PVL) and functional assays (ClfA, Hla, LukAB and SpA), comparison between ISA and HC samples was performed using Welch’s Student’s t-test with multiple testing correction (Benjamini-Hochberg procedure).

## Results

### Genomic Analysis of ISA Isolates Shows a Variability Similar to the Reference Strains

**Figure 1:**
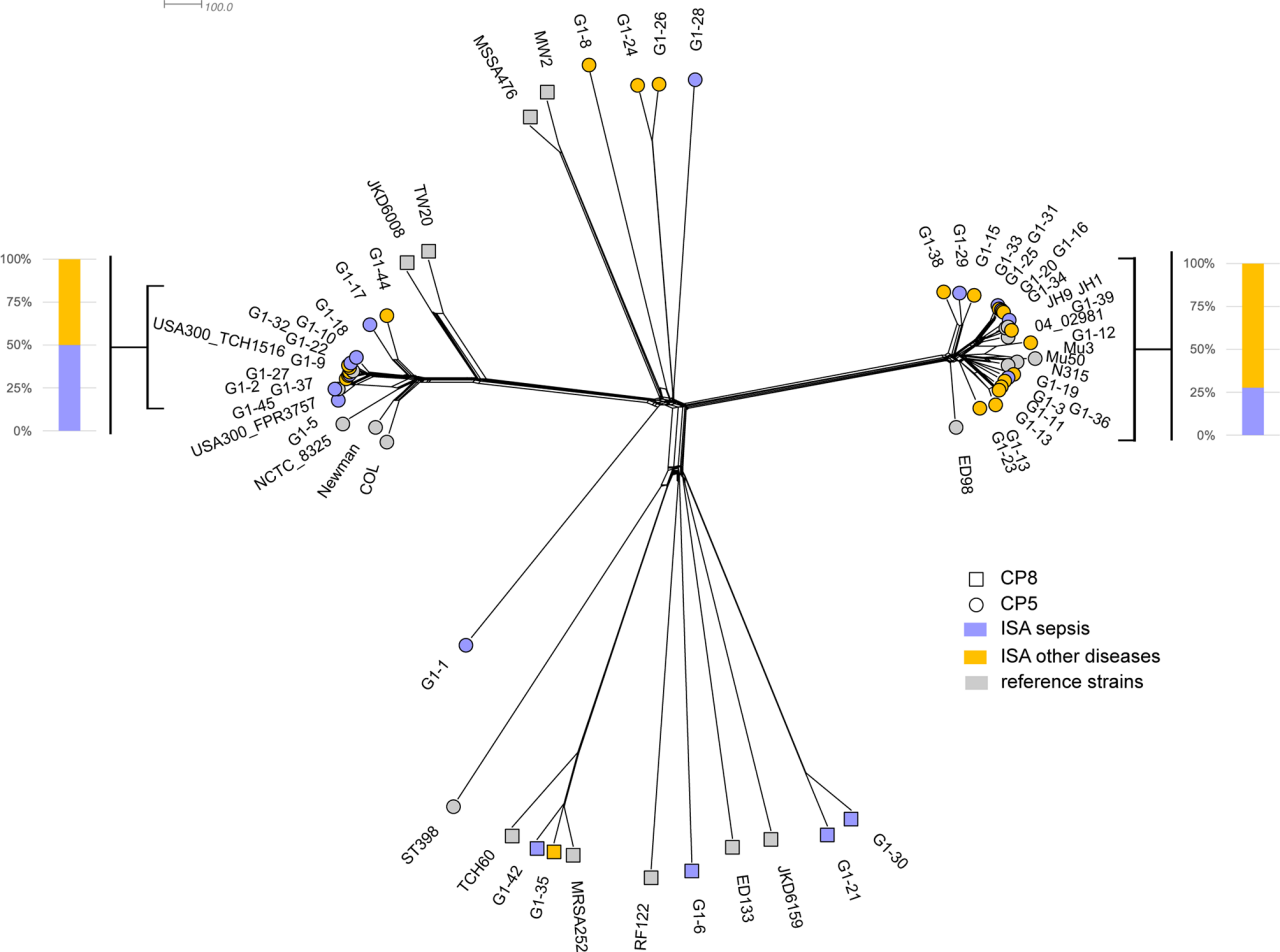
The neighbor phylogeny based on the variability of MLST genes + 2208 species core genes. Violet and yellow tips indicated strains from patients in ISA group with sepsis and other primary diseases, respectively. Grey tips indicated the 22 reference strains.

The genomic variability of infecting isolates was compared to a dataset of 22 genomes representative of *S. aureus* species. The isolates collected in the study were genetically diverse and were representative of circulating strains; the majority of isolates in the ISA collection form clusters with the reference strains (**Figure 1**). The prevalent sequence type is ST-8, followed by ST-5, and ST-105 (data not shown). Capsular polysaccharide genes were also characterized showing that strains isolated from patients with invasive disease preferentially encoded CP5 locus (35 isolates carrying CP5 and 5 carrying CP8).

The phylogenetic analysis confirms that diverse isolates can be responsible for different invasive disease manifestations and isolates causing sepsis (17 isolates) and other primary diseases (23 isolates) are distributed along the entire phylogenic tree. Two major compact (clonal) clades were identified, clustering respectively with USA300 strains (USA300_FPR3757 and USA300_TCH1516) and with Mu3/Mu50 strains (on the left and on the right of the phylogenetic tree in **Figure 1**, respectively). Although the relative percentages of isolates causing sepsis or other diseases differs in the two clades, this imbalance is not statistically significant (p-value > 0.26 in Fisher’s exact test).

Moreover, the two clades encode exclusively CP5, whereas CP8 isolates are spread in the phylogeny and they show a higher degree of diversity.

### Circulating Cytokines Show a Differential Pattern in *S. aureus* Invasive Disease

**Figure 2:**
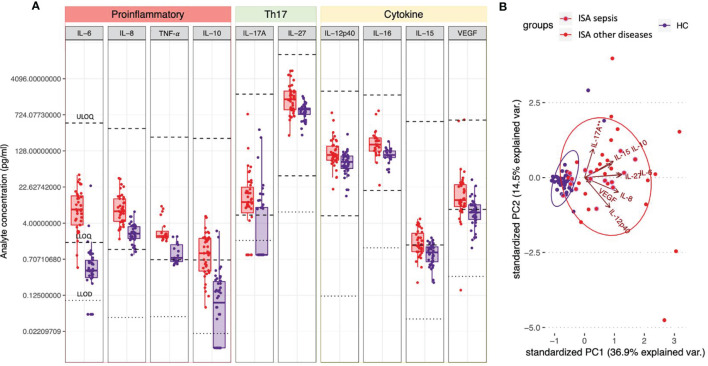
Circulating cytokines defining a S. aureus invasive disease signature. **(A)** Plasma or serum samples obtained from patients with invasive S. aureus disease (ISA, in red) or from healthy controls (HC, in purple) were tested for the presence of cytokines/chemokines by 36-V-PLEX assay. Individual concentrations are represented as dots. Boxplots represent first and third quartile; median, minimum and maximum values are also shown. For each analyte light dotted lines represent lower limits of detection (LLOD) and bold dotted lines represent upper and lower limits of quantification (ULOQ and LLOQ). Concentrations below detection or fit curve range were assigned a concentration corresponding to ½ LLOD. Analytes’ concentrations detected in ISA *vs.* HC were compared by Dunn’s post-test after Kruskal-Wallis non-parametric test. Only analytes for which P<0.05 and at least the median of one of the two groups analyzed was in the quantification range are shown. **(B)** Principal Component Analysis (PCA) of the cytokines shown in A. Red dots and ellipses refer to ISA while blue ones refer to HC. Among the ISA samples (in red), the sub-group relating to patients with sepsis are circled in purple. Arrows reveal how each cytokine contributed to the PCA plotted in the graph.

To identify cytokines and chemokines that are involved in the immune responses to invasive *S*. *aureus* disease, we determined the concentrations of specific analytes in the samples collected during the infection and compared them to those detected in healthy donors. As shown in **Figure 2A**, patients with invasive disease exhibited statistically higher concentrations of 10 out of the 26 measured analytes as compared to healthy controls. In particular, concentrations (pg/mL) of IL-6 (7.68 *vs.* 0.41), IL-8 (7.04 *vs.* 2.41), TNF-α (2.14 *vs.* 0.75), IL-10 (0.94 *vs.* 0.09), IL-17A (10.94 *vs.* 0.87), IL-27 (1522.37 *vs.* 933.69), IL-12p40 (105.09 *vs.* 75.37), IL-16 (173.09 *vs.* 104.91), IL-15 (1.39 *vs.* 0.98), and VEGF (12.05 *vs.* 6.65) were increased in ISA patients (see **Supplementary Table 1** for complete dataset). Overall, a mild pro-inflammatory response was observed (higher concentrations of IL-6, IL-8, TNF-α, IL-16, IL-15, VEGF), together with an increase of immunomodulatory cytokines (IL-10, IL-27, and IL-12p40). Remarkably, while circulating IL-17A was increased overall and above the lower limit of quantification (LLOQ, 5.86 pg/mL) in the majority of patients with invasive disease but not healthy controls (74.4% *vs.* 33.3%), IFN-γ levels were low (<LLOQ, 7.47 pg/mL) and above the LLOQ only in 33.3% of patients, while IL-4 and IL-13 were undetectable in both groups (<LLOD, 0.01 pg/mL), indicating a type-17 kind of response in ISA patients.

We also performed principal component analysis (PCA) using analytes that significantly differed between the two groups. PCA clearly discriminated infected subjects (ISA) from healthy controls (HC) (**Figure 2B**, red and blue ellipses, respectively); of note, only six ISA samples were not separated from the HC samples. The analysis revealed that the first component in the separation was primarily influenced by IL-8, IL-27, IL-6, IL-15 and IL-10, accounting for 36.9% of the variance. The second component, primarily influenced by IL-12p40 and IL-17A, accounted for an additional 17.5% of the variance. The HC group showed homogenous responses, whereas ISA subjects showed scattered levels of cytokines production, likely due to the individual differences in the response to the infection and the diverse timing of sample collections. Moreover, the pattern of the cytokines in the patients with sepsis and other primary diseases showed a similar distribution.

### Patients With Invasive Staphylococcal Disease Demonstrated a Distinctive Antigen-Specific Immune Response by Protein Array Analysis

To identify an immune signature capable of differentiating the immune responses of infected patients from healthy controls, we performed a high throughput protein array analysis targeting 104 surface-expressed or secreted antigens (see **Supplementary Table 2** for complete list of antigens with the respective locus_tag, GI number, annotation and Psort-B prediction). We identified a panel of 24 best recognized staphylococcal antigens; importantly, the reactivity levels against the majority of them (15 out of 24) was significantly higher in patients with invasive *S. aureus* disease compared to healthy controls (**Table 1**).

**Table 1:** Best recognized *S. aureus* antigens in protein chip analysis.

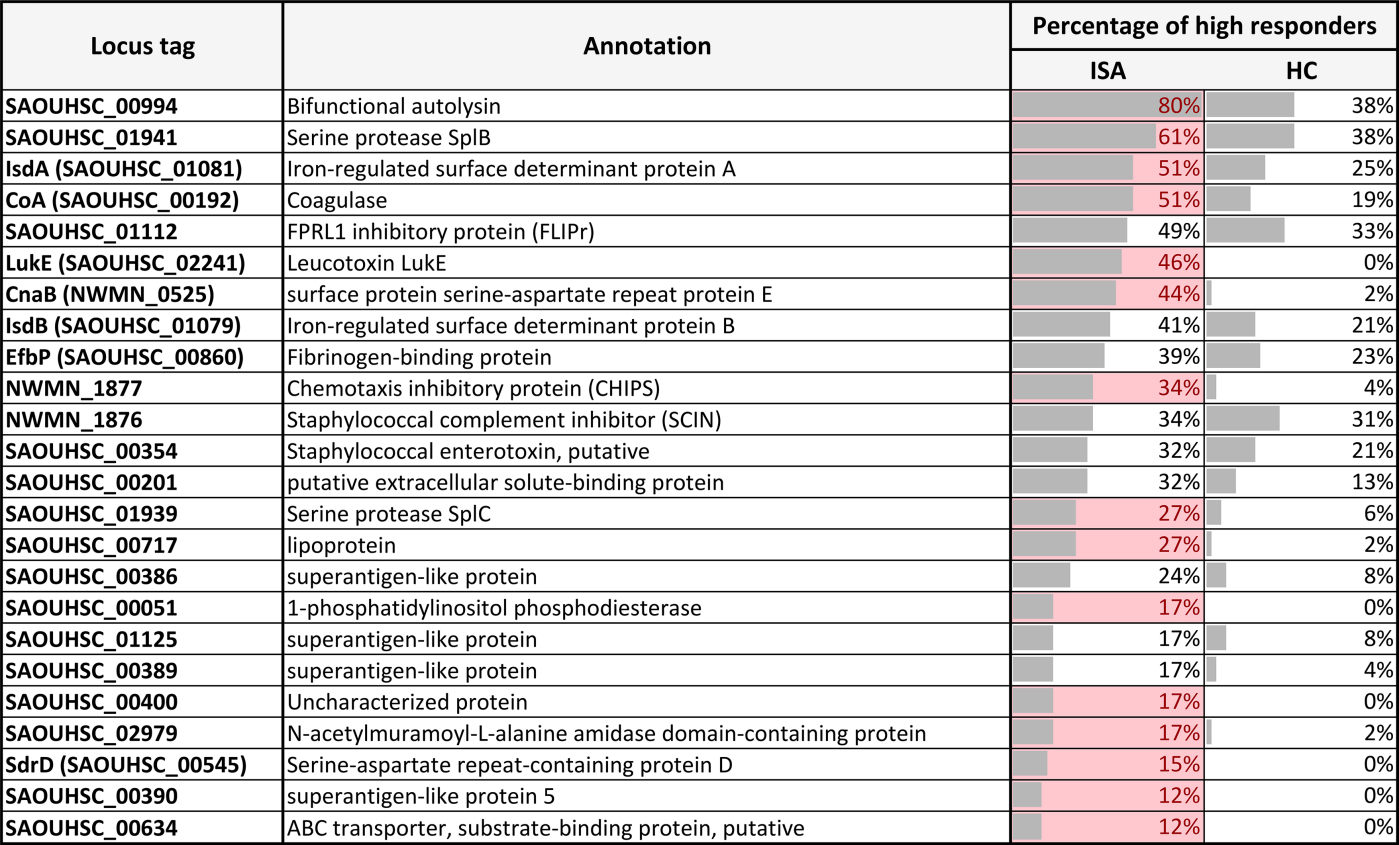 )

For each protein the percentage of high responders in invasive disease (12) and healthy (HC) subjects is reported. Only proteins with a concentration above 0.05 in at least 10% of the ISA subjects were reported in the table. Proteins for which the high responders of ISA are significantly higher than HC (P< 0.05) are highlighted in red.

**Figure 3:**
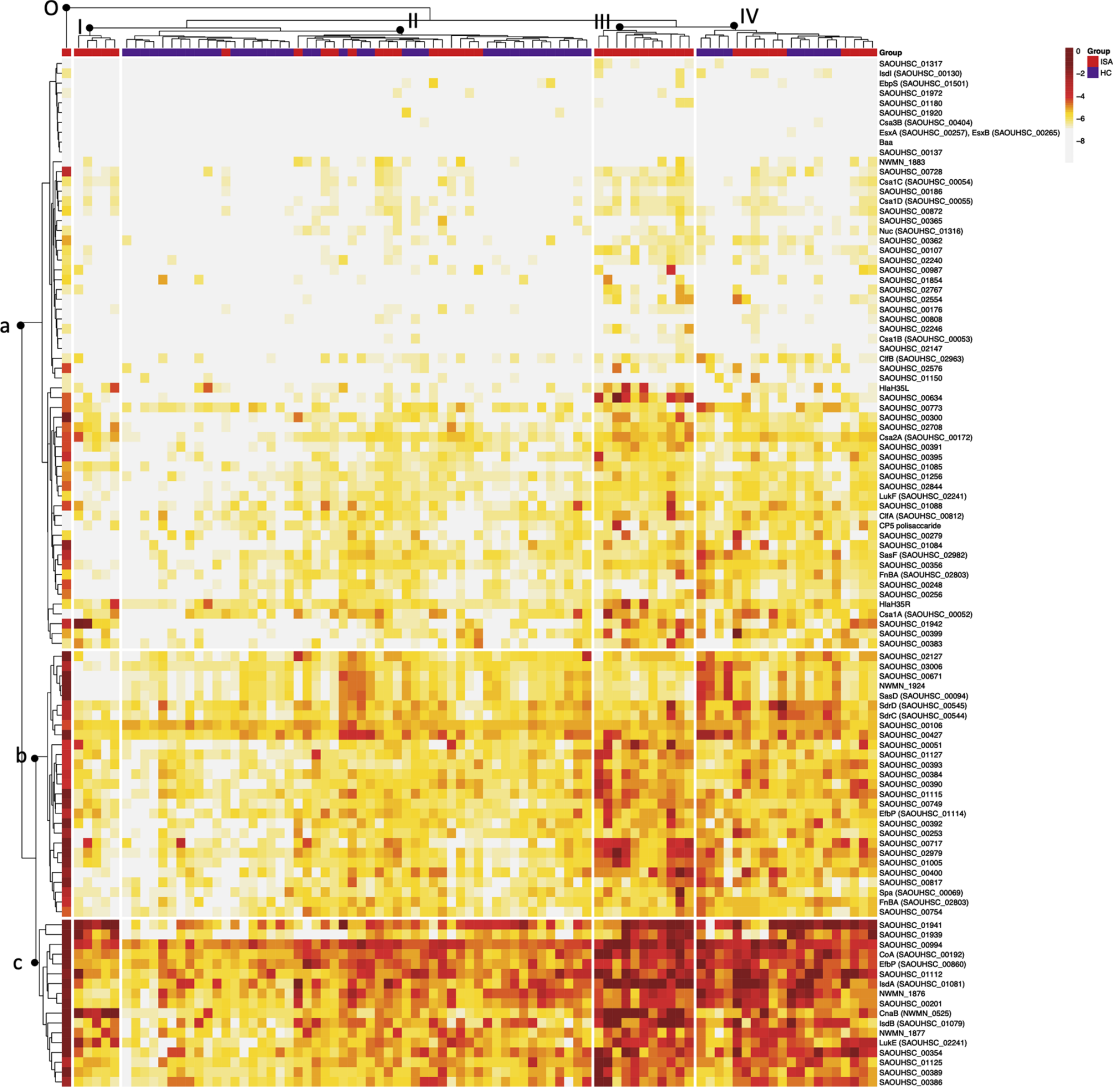
Heatmap and bidimensional cluster analysis of all normalized data based on Euclidean distance. Cluster analysis of the Mean Fluorescence Intensity (MFI) data obtained from all S. aureus antigens tested (rows) against single sera sample (columns). Darker to lighter tones indicate the concentrations of each sample and protein as indicated in the scale bar. The five major clusters identified in the sera are indicated on top of the dendrogram (I-II-III-IV; O: outlier cluster). For each subject are reported in the clustering the corresponding group, depicted in red (12) and in blue (HC). The three major clusters are indicated with a dendrogram on the left-hand side of the graph (a = low, b = medium, c = high).

Heatmap and bidimensional cluster analysis revealed the presence of three groups of antigens, with, low (a), medium (b) and high (c) reactivity (dendrogram on the left side of the heatmap in **Figure 3**). The three groups of reactivity showed no specific clustering of antigens based on defined biological characteristics, as they are all linked to *S. aureus* infection and have extracellular localization. We observed in medium and low reactive groups an enrichment of membrane antigens and antigens involved in cell adhesion, as annotated through a functional annotation tool (https://david.ncifcrf.gov). The largest group of antigens was the one at low reactivity, indicating that most of the genes were not activated. Unsupervised clustering of patients, represented in , was coherent with clinical characteristics, separating ISA patients from HC. Four main groups could be identified (**Figure 3**); the major difference was between group III which included exclusively ISA patients, showing generally higher reactive immunosignatures, and group II, with lower reactive profiles, which included ISA patients and healthy controls. Interestingly, we identified an outlier sample from a patient with epidermolysis bullosa, which exhibited the highest reactivity (group 0 in the left side of the heatmap).

### Analysis of the Host Response to Key Virulence Factors Revealed an Invasive-Specific Antibody Response Against Staphylococcal Toxins

**Figure 4:**
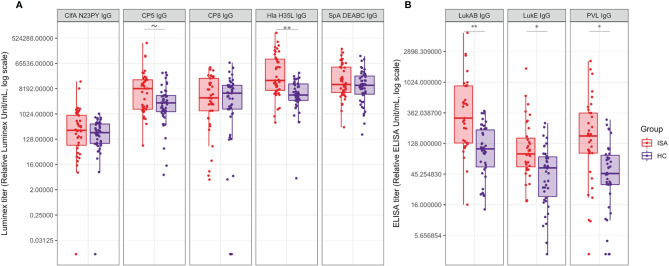
Antibody titers against a panel of S. aureus virulence factors. Boxplot and scatterplot are reported to summarize IgG quantification levels by Luminex or ELISA. **(A)** reports the IgG values for ClfA, CP5, CP8, Hla and SpA, measured by Luminex analysis; **(B)** reports the IgG values for LukAB, LukED and PVL measured by ELISA. Samples in ISA group and HC are indicated in red and blue, respectively. Tilde and stars on top of the boxes report the level of significance of the Welch’s t-test p-values on the means: ~ 0.052, * < 0.05, ** < 0.001.

We measured serum IgG levels against ClfA, CP5, CP8, Hla, SpA, LukAB, LukED and PVL, known to be relevant virulence factors contributing to *S. aureus* pathogenesis (**Figure 4**).

ClfA showed particularly low titers, with similar distribution between ISA and HC groups. Similarly, anti-CP8 and anti-SpA IgG titers antibodies showed comparable distributions in the two groups. On the contrary, antibody titers against CP5, Hla, LukAB, LukED and PVL were significantly higher in ISA patients than in HC.

**Figure 5:**
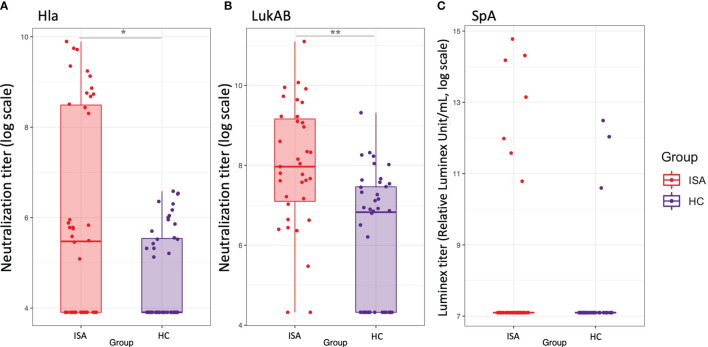
Functional antibodies against Hla, LukAB and Spa. Boxplot and scatterplot are reported to summarize functional antibodies quantification levels. **(A, B)**, report the values of neutralization titers for Hla and LukAB toxins, respectively, defined as the reciprocal serum dilution which neutralizes the toxicity by 50%. **(C)** reports the values of functional titers for SpA binding to FcgRI, as calculated by a Luminex-based measurement. Samples in ISA group and HC are indicated in red and blue, respectively. Stars on top or bottom of the boxes report the level of significance of the Welch’s t-test p-values on the means: *< 0.05, **< 0.001.

We then focused on the functional analysis of antibodies measuring the ability to inhibit the pathogenic effects of ClfA, Hla, SpA, LukAB. Hla-specific functional antibodies, able to neutralize hemolysis induced by α-toxin, were higher in patients with ISA as compared to HC (**Figure 5A**). Similarly to Hla, higher neutralizing LukAB antibodies able to inhibit the toxic effect on neutrophil viability, were found in sera from invasive disease patients, compared to the healthy controls (**Figure 5B**). For both families of toxins there was correlation between antibody levels and their functionality, as measured by univariate regression analysis (data not shown).

Functional antibodies against SpA, defined as the ability to bind to the Fab portion of the antibodies, leaving the Fc portion available for the binding to soluble FcγRI, were detectable only in few subjects; interestingly high levels of functionality were detected in four samples from the infected group (**Figure 5C**). Finally, in agreement with previous observations (15), ClfA was poorly immunogenic showing only one serum sample with weak inhibitory activity (data not shown).

## Discussion

*S. aureus* colonizes a high proportion of healthy subjects and can cause a wide range of clinical phenotypes and disease severity. These multifaceted phenotypes highlight the importance to identify signatures which can discriminate between the pathogenic and the carrier status. In the present study, we aimed to answer this key question interrogating the host-pathogen interplays by different angles: we performed a genome analysis of bacterial isolates to define the genetic diversity of the causative strains, we analyzed the innate immune response to the infection, and we dissected the adaptive responses through both a whole analysis of antibody repertoire and an antigen-specific examination.

The study covers patients with diverse invasive *S. aureus* infections, such as bacteremia, endocarditis, osteomyelitis, and disseminated/multi-focal diseases, offering the opportunity to analyze a range of different primary diseases.

Genomic analysis of strains isolated from each patient revealed no major differences in genetic distribution compared to a panel of circulating strains. The strains isolated from patients with sepsis or other primary diseases, showed a high heterogeneity and they were spread in the entire phylogeny without grouping in clusters associated to the kind of disease. Genotyping of the capsule was also carried out, showing that most of the strains belong to CP5 genotype (87%) and the majority of them were grouped in two major clades genetically distant and associated with USA300 and Mu3/Mu50 strains, respectively. Of note, USA300 clonal lineage does not produce a capsular polysaccharide due to conserved mutations in the CP5 locus (16). *S. aureus* can express 13 putative capsular polysaccharides; however only isolates that express capsular polysaccharide type 5 (CP5) or 8 (CP8) have been associated with disease (17) and are produced by 75–80% of *S. aureus* clinical isolates (18). An epidemiological study on capsular polysaccharides in clinical isolates in the United States showed that CP5 genotypes were the most prevalent (19). The prevalence of CP5 in the isolates described in the current study is then consistent with the documented capsular epidemiology.

The analysis of the inflammatory responses revealed a panel of circulating cytokines distinctive of ISA patients as compared to HC. Notably, pro-inflammatory cytokines, like IL-6, IL-8, and TNF-α, and IL-17A were significantly higher in the patients with invasive disease. The differential cytokines levels in invasive disease and their correlation with clinical outcomes has been matter of several studies [reviewed in (6)]. Survival and a less complicated course of infection correlated with early rise of low levels of pro-inflammatory cytokines including TNF-α (20). Overall, the mild pro-inflammatory responses measured in this study are consistent with the favorable outcome of invasive *S. aureus* disease in the enrolled patients in which no fatal outcomes were observed. Extracting the pattern of the cytokines in the patients with sepsis, we found that TNF-α has a bi-modal distribution, having either very high or very low levels (data not shown). Moreover, the sub-set of the patients with sepsis had low levels of IL-27, which was reported to correlate with improved outcome (20). Also, high levels of IL-6, IL-8, and IL-10 have been previously associated with more complicated disease manifestations (12, 21). Overall, our results partially align with previous findings. Variations among the studies may be due to the timing of sampling, since patients are enrolled in the study in different stages of infection and received different treatments. The samples were taken after culture diagnosis of *S. aureus* infection and, depending on the timing, some early rise of acute cytokines could have been missed in the analysis. On the other hand, differences may be due to the various clinical patterns of disease in our study as compared to previous studies mainly conducted in patients with sepsis.

The pathogenesis of *S. aureus* is mediated by a panoply of virulence factors that intervene at various levels in the interaction with the host. Previous studies have detected circulating antibodies against surface-exposed and secreted antigens in healthy population (7). Specifically, nasal colonization appears to induce antibodies with higher levels in persistent carriers than in non-carriers (8, 9). The most comprehensive previous study analyzed the levels of serum IgG and IgA antibodies against 56 staphylococcal antigens from 21 patients with *S. aureus* bacteremia, showing that serum IgG levels against 27 antigens were significantly higher in bacteremia patients, compared with non-infected controls (22).

In the attempt to identify an antibody repertoire distinctive of invasive disease, we investigated the antibody response to wide panel of antigens, and also the ability of the antibodies to be functional against a panel of antigens known to be important in the pathogenesis. By a high-throughput microarray analysis on 104 antigens selected by in silico analysis combined to proteomic data (13), we observed a wide responsiveness to the pathogen from all subjects of the study both in HC and ISA groups. Indeed, the majority of the sera from both groups showed a detectable reactivity to most of the antigens in the array repertoire. However, when we applied a hierarchical clusterization and principal component analysis based on antibodies level, we found a clear separation between patients with invasive diseases and healthy subjects. The majority of the most activated antigens are significantly higher in the invasive patients compared to control. Heatmap and bidimensional cluster analysis showed four clusters of responsiveness were the distribution between ISA and HC are either equal (cluster IV) or specific for invasive status (cluster III). Overall, these data indicate that the level of responsiveness in the healthy population is variable and baseline levels of antibody response are substantially different in the cohort analyzed in this study. None of the patients enrolled in this study had severe complications or mortality, and consequently no association between antibody pattern and severity of disease can be asserted. However, these findings suggest that high levels of an antigen-specific subset of antibodies could represent a signature for systemic infections.

While the protein array gave us a comprehensive view on the serum profiling following systemic infections, the specific immune response to key virulence factors was examined in depth by the analysis of antibodies levels and functionalities, employing specific functional assays tailored on virulence activity. Interestingly, antibody response against ClfA, SpA and CP8 showed comparable distribution in the invasive and healthy groups, with a minor trend of higher CP5-specific antibody titers in ISA patients. This latter evidence is consistent with the evidence that the majority of the isolates preferentially encoded CP5.

In contrast, antibody response against toxins such as Hla, leukocidins and PVL distinctively associate with the invasive disease. Analyzing the data with a separation between the patients with sepsis and the ones with other primary diseases, we found similar distribution in Hla and LukAB functional antibodies (data not shown).

The responses against Hla have been previously reported and high antibody titers against the toxin are generated following *S. aureus* invasive infections, including bacteremia and pneumonia (23, 24). Similarly, LukAB response is frequent in invasive disease and children with diagnosis of *S. aureus* disease generate high serum anti-LukAB titers early in the course of disease (25), leading to the evidence that LukAB antibodies might be useful in the diagnosis of invasive bacterial infections (26). A possible explanation for the evidence that functional antibody responses against toxins are distinctive of *S. aureus* invasive disease is that surface-associated antigens are relatively protected by complex immune evasion mechanisms on the surface of the organism. Secreted toxins may also be more expressed and thus more exposed to the host immune system during invasive disease, resulting in a targeted, protective disease-specific antibody response.

In conclusion, we reported the evidence of specific signatures of cytokines and protective antibodies in patients with different primary invasive diseases caused by *S. aureus*. Although the sample size may not allow full stratification by different diseases phenotypes and possible associations therein, these findings suggest that specific patterns of cytokines and antigen-specific antibodies may represent a signature for systemic infections and provide further insight into invasive *S. aureus* pathogenesis and host responses.  

## Data Availability Statement

The raw data supporting the conclusions of this article will be made available by the authors, without undue reservation.

## Ethics Statement

The studies involving human participants were reviewed and approved by VUMC Human Subjects Protection Program (IRB). Written informed consent to participate in this study was provided by the participants’ legal guardian/next of kin.

## Author Contributions

RL wrote the manuscript and performed the ClfA assay. MB made the statistical analysis and wrote the manuscript. IT conceived and designed the study and wrote the manuscript. ES performed cytokine assay and analyzed the data. EBa performed protein chip experiments and analyzed the data. AM performed genomic analysis and analyzed the data. BC performed cytokine assay. BG performed Luminex assay. AGOM performed SpA assay. CG performed Hla assay. SC sequenced the strains. SB made the statistical analysis. FS performed Luminex assay. EBo performed cytokine assay. SR and IM contributed to data interpretation. FB conceived the study. GG and CC conceived the study and wrote the manuscript. All authors revised the manuscript and approved the submitted version.

## Funding

The study was sponsored by Vanderbilt University Medical and Novartis Vaccines and Diagnostics Srl (in 2015, Novartis’ non-influenza vaccines business was acquired by GlaxoSmithKline Biologicals SA).

## Conflict of Interest

All authors except GG, SC, IT, and CC are employees of the GSK group of companies. FB and IM hold shares and/or restricted shares in the GSK group of companies. GG was employed by the GSK group of companies during the conduct of this study and held stock options and stocks in the GSK group of companies as part of his employee remuneration. SC was employed by the GSK group of companies during the conduct of this study. FB holds pending and issued patents on *S. aureus* vaccine formulations. IM and RL are listed as inventor on patents owned by the GSK group of companies. BC has non-financial support from the University of Siena outside the submitted work. The authors declare that the research was conducted in the absence of any commercial or financial relationships that could be construed as a potential conflict of interest.

The authors declare that this study received funding from Novartis Vaccines and Diagnostics Srl. The funders had the following involvement with the study: study design, conduct, analysis and data interpretation.

## Publisher’s Note

All claims expressed in this article are solely those of the authors and do not necessarily represent those of their affiliated organizations, or those of the publisher, the editors and the reviewers. Any product that may be evaluated in this article, or claim that may be made by its manufacturer, is not guaranteed or endorsed by the publisher.
