# Supplementary material for: Dissecting the Human Response to Staphylococcus aureus Systemic Infections
Source: Front Immunol. 2021 Nov 8;12:749432. doi: 10.3389/fimmu.2021.749432 (PMC8607524; doi:10.3389/fimmu.2021.749432)
Supplement: Supplementary file 1 [file DataSheet_1.docx]

Supplementary Material


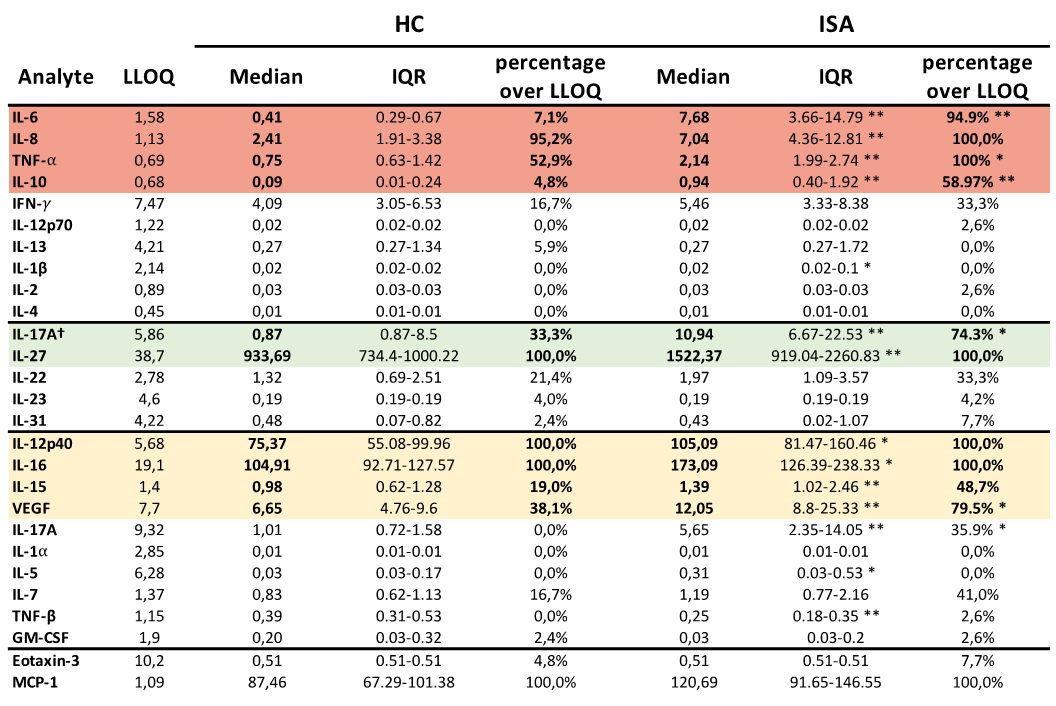


**Supplementary Table 1.** Circulating cytokines levels are altered in S. aureus invasive disease. Plasma or serum samples obtained from 42 healthy controls (HC) or from 39 patients with invasive S. aureus disease (ISA) were tested for the presence of cytokines/chemokines by 36-V-PLEX assay. For each analyte the lower level of quantification (LLOQ), the median, inter quartile range (IQR) and percentage of observations over the LLOQ in the two groups are reported. Stars in the IQR and percentage over LLOQ columns indicate the level of significance of the Dunn’s test and the test of proportions, respectively: * < 0.05, ** < 0.001. The analytes showing significance in the comparison between ISA and HC and at least one of the two medians over the LLOQ are highlighted with different colors according to the 36-V-PLEX assay test plate. †: IL-17A GenB.

**Supplementary Table 2**. List of 104 antigens belonging to the *S. aureus* strain NCTC 8325 or Newman, produced in *E. coli* as recombinant proteins. For each antigen the respective locus_tag, GI number (GenInfo Identifier assigned by NCBI), product name and Psort-B prediction are indicated.
